# The heat is on: Consumers modify their oral processing behavior when eating spicy foods

**DOI:** 10.1016/j.crfs.2023.100597

**Published:** 2023-09-29

**Authors:** Cong Lyu, Marlotte Vonk, John E. Hayes, Jianshe Chen, Ciarán G. Forde, Markus Stieger

**Affiliations:** a Division of Human Nutrition and Health, Wageningen University, P.O. Box 17, 6700 AA Wageningen, the Netherlands; b Sensory Evaluation Center, Department of Food Science, College of Agricultural Sciences, The Pennsylvania State University, 220 Food Science Building, University Park, PA, 16802, USA; c Laboratory of Food Oral Processing, School of Food Science and Biotechnology, Zhejiang Gongshang University, Hangzhou, China; d Food Quality and Design, Wageningen University, Wageningen, the Netherlands

**Keywords:** Food oral processing, Eating behavior, Chili pepper intake, Chemesthesis, Rate-all-that-apply

## Abstract

Food texture properties and consumer characteristics influence oral processing behaviors. Little is known about oral processing behavior of pungent spicy foods. In two experiments, we investigated how adding ground dried chilies to tomato soup or beef patties and curried rice altered oral processing behaviors. In Experiment One, tomato soups differing in concentration of added ground dried chilies (0.01, 0.03, 0.20 or 0.40% w/w) were consumed (n = 23). In Experiment Two, lunch meals that differed in added ground dried chilies consisting of beef patties (0.0, 0.6 or 1.2% w/w) and curried rice (0.0, 0.4 or 1.0% w/w) were consumed (n = 49). Sip/bite sizes were determined using hidden balances. Oral processing behavior was quantified using video recordings followed by post hoc annotations of specific behaviors. When eating tomato soup, increasing oral burn was associated with increasing number of water sips, water intake and total time between sips. For the solid meals (beef patties and curried rice), increasing oral burn was associated with increased time between bites and total sips of water; conversely, total oral exposure time, total number of chews and number of chews per bite all decreased with greater burn. Saliva content and rate of saliva incorporation into the solid food bolus increased with added ground dried chilies while oral exposure time decreased. We conclude consumers adapt their oral processing behaviors to oral burn of solid foods by reducing oro-sensory exposure time, chewing bites less, increasing time between bites, and consuming more water, potentially to mitigate the discomfort associated with the burn imparted by ground dried chilies.

## Highlights

• Consumers adapt their oral processing behaviors to oral burn when eating spicy foods.
• Increased burn of tomato soups increased number of water sips, water intake and total time between sips.
• Increased burn of beef patties and curried rice decreased oral exposure time, number of chews and number of chews per bite.
• Consumers reduce oro-sensory exposure time, chew bites less, increase time between bites, and consume more water.

## Graphical abstract

**Figure:**
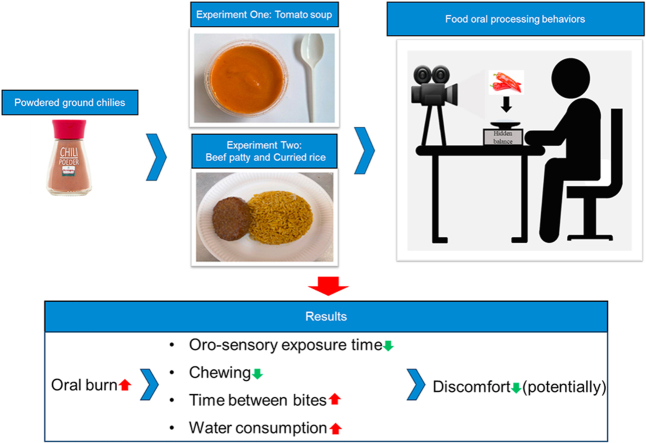

## 1 Introduction

Food oral processing is the dynamic process of food breakdown during mastication that can influence digestion and sensory perception of foods (Chen, 2009). Numerous studies have investigated oral processing behaviors for solid foods, finding large variations in these behaviors depending on the mechanical, rheological, texture and tribological properties of foods and the characteristics of the consumer (Ferriday et al., 2016; Forde et al., 2017, Forde et al., 2013b, Forde et al., 2013a).

Physical-chemical, texture, mechanical and tribological properties of foods are known to influence oral processing behaviors (Aguayo-Mendoza et al., 2019; Chen and Stokes, 2012; Devezeaux de Lavergne et al., 2017; Hiiemae, 2004). For example, consumption time and number of chews are associated with mechanical and texture properties such as fracture stress, fracture strain, Young's modulus and adhesiveness for a broad range of solid foods (Barrangou et al., 2006; Çakir et al., 2012; Foster et al., 2006; Jourdren et al., 2016; Koç et al., 2014; Panouillé et al., 2014; Pascua et al., 2013; Peyron et al., 2002; Wagoner et al., 2016; Wee et al., 2018). Increasing fracture stress of model food gels prolongs consumption time, and increases number of chews, muscle activity and jaw opening amplitude. Eating rate depends strongly on texture properties for a broad range of foods (Forde et al., 2013a; 2013b; McCrickerd et al., 2017). Bolhuis et al. (2014) demonstrated that the eating rate of hard solid foods was 32% lower than that of soft solid foods. Separately, consumer characteristics such as age, gender and ethnicity also influenced oral processing behaviors of a broad range of liquid, semi-solid and solid foods (Ketel, et al., 2019; Ketel et al., 2020). Aging impacts oral processing behaviors and is linked to a decrease in dental status, reduced salivation and declined facial muscle strength (Affoo et al., 2015; Alsanei and Chen, 2014; Kohyama et al., 2002). Sex has been shown to affect oral processing behaviors with males consuming foods with larger bite size and shorter consumption time, which leads to a faster eating rate relative to females (Ketel et al., 2019; Park and Shin, 2015).

In contrast to numerous studies on food texture, mechanical and lubrication properties and consumer characteristics and oral processing behavior, only a few studies have examined the influence of taste or aroma on oral processing behaviors. Regarding taste, it has been demonstrated that higher salt intensities were associated with fewer sips and smaller sip sizes in tomato soups (Bolhuis et al., 2011, 2012). A higher salt intensity in chewing gums led to a shorter chewing time (Neyraud et al., 2003). Neyraud et al. (2005) observed shorter mastication times (fewer bites, less muscle effort) and longer clearance times with increasing bitterness of model gels, while Alfonso et al. (2002) failed to find an effect of bitterness on chewing muscle activity and chewing frequency for model gels. Forde et al. (2013b) suggested that eating rate of lunchtime meals did not differ between high savory and standard savory meals whereas others have shown that neither number of chews, chewing duration, chewing rate, nor eating rate were affected by the sweetness intensity of model gels (Lasschuijt et al., 2017). Recently, Gonzalez-Estanol et al. (2022) showed that large differences in sweetness and saltiness intensity did not affect oral processing behaviors of liquid (tomato and strawberry sauce), solid (penne pasta and milk gel) and composite (penne pasta + tomato sauce and penne pasta + milk gel) foods while food consistency and lubricity did. Collectively, these studies indicate oral processing behaviors are primarily driven by texture, mechanical and lubrication properties of foods rather than by taste quality or intensity. Similarly, the influence of aroma on oral processing behaviors has a modest effect. With studies showing the a higher aroma intensity in a vanilla custard resulted in smaller average bite size (de Wijk et al., 2012). Other studies suggest that mastication rate, number of chews and chew amplitude are positively correlated with aroma concentration for a variety of foods (Haahr et al., 2004; Hansson et al., 2003; Mestres et al., 2006; Pedrotti et al., 2019; van Ruth and Roozen, 2000).

Little is known about how chemesthetic sensations, like the oral burn from capsaicin, influence the microstructural patterns of eating. Capsaicin is a chemesthetic compound found in chili peppers which are used in many cuisines around the world for the flavor they add to dishes. Capsaicin is a secondary plant metabolite that prevents ingestion by most mammals, with the marked exception of humans, who acquire a preference for its chemesthetic properties and have sought out its burn for millennia. Typically, the affective dose-response curve is an inverted U-shaped curve (Wundt Curve, inverted parabolic/bell-shaped curve) in humans: liking first increases and then declines as concentration increases (Lyu et al., 2021; Nolden and Hayes, 2017). As an oral irritant, capsaicin is known to stimulate salivary glands and increase saliva secretion (Kanehira et al., 2011; Nasrawi and Pangborn, 1990; Yang et al., 2020), which in turn may influence oral processing behaviors. Greater saliva secretion may lead to increased saliva uptake in the food bolus, thereby facilitating more rapid agglomeration and the formation of a safe-to-swallow bolus in a shorter mastication time. Recently, Djekic et al. (2021) reported that hot sauces added to pork increased salivation without significantly affecting oral processing behaviors. More studies are needed to understand how consumers adapt their oral processing behaviors when hot spicy foods are consumed. A prior meta-analysis found weak evidence that consuming capsaicin before a meal may slightly reduce the amount of food consumed and might drive food preference towards carbohydrates (Whiting et al., 2014). We speculate that this may be due to changes in eating rate at which foods are consumed which is well-known to impact food and energy intake (Bolhuis and Forde, 2020; Forde and Bolhuis, 2022; Robinson et al., 2014). If this is the case, adding hot spicy condiments to food may afford new opportunities to modify eating behaviors and food intake without manipulating food texture properties.

The primary aim of the present study was to investigate the influence of oral burn from chili peppers on oral processing behavior of liquid (tomato soup) and solid (beef patties and curried rice) foods. Dried ground chilies were added to soups, beef patties and curried rice at different concentrations to vary the intensity of oral burn. For practical and logistical reasons, we performed two experiments sequentially (Experiment One: tomato soup; Experiment Two: lunch meal consisting of beef patties and curried rice). The two experiments used different sensory methodologies to characterize the stimuli and different participants but had comparable aims. We hypothesize that (i) oral burn caused by addition of ground chilies influences oral processing behaviors of soups, beef patties and curried rice. Specifically we hypothesized that (ii) bite/sip size, number of chews/sips, number of chews per bite, oral exposure time and eating rate decrease with increasing oral burn intensity as consumers adapt their oral processing behaviors to minimize discomfort.

## 2 Materials and methods

### 2.1 Participants

Participants of varying races and ethnicities were recruited from Wageningen campus and surroundings using social media and a database of volunteers with an interest in human studies of Wageningen University & Research (WUR) in the Netherlands. Interested individuals were selected after undergoing a screening session and met the following inclusion criteria: 18–60 years old, having complete dentition, no chewing or swallowing problems, BMI of 18.5–30 kg/m^2^, being willing to eat (moderately) spicy foods, no food allergies for any of the food products used in this study, no energy-restricted diet or having a weight change of more than 5 kg in the last two months, not pregnant or intentions to become pregnant, not breastfeeding, not taking any medication that may affect the function of taste, smell, mastication or salivation, and non-smoker. Participants meeting these criteria completed the chili pepper questionnaire to assess their liking and habitual intake of a variety of foods containing chili peppers (Byrnes and Hayes, 2013; 2016; Choi and Chan, 2015; Lawless et al., 1985; Lyu et al., 2021; Nolden and Hayes, 2017; Reinbach et al., 2007). This information was used for post-hoc comparisons of the impact of frequency of chili consumption on each individual's observed oral processing behaviors in the current study.

A total of 23 participants (17 females and 6 males, 25.0 ± 3.3 years, BMI 22.6 ± 2.8 kg/m^2^) participated in Experiment One, and a total of 49 participants (31 females and 18 males, 26.3 ± 8.2 years, BMI 21.8 ± 1.8 kg/m^2^) completed Experiment Two. To reduce the impact of learning, participants were only allowed to participate in only one of the experiments, not both. Experiment One was an exploratory proof of concept pilot; power was increased in Experiment Two by recruiting more participants. Written informed consent was obtained from participants prior to participation, and they were free to withdraw at any time, in accordance with the Declaration of Helsinki, as updated in 2013. All participants were reimbursed for their participation. The study did not meet the requirements to be reviewed by the Medical Research Ethical Committee of The Netherlands according to the “Medical Research Involving Human Subjects Act” of The Netherlands (WMO in Dutch).

### 2.2 Materials

Powdered ground chilies (100% chili pepper, Verstegen Spices & Sauces) was purchased in a local supermarket. Varying amounts of ground chilies were added to test foods to evoke different burn intensities in Experiment One and Two. Pilot testing was used to achieve moderate and strong oral burn without being so strong that participants would stop consuming the meals. During pilot testing, a broad range of concentrations of ground chilies were added to the food matrices, and oral burn intensity was rated by 12 participants (6 women and 6 men). A general Labelled Magnitude Scale (gLMS) was used to rate burn intensity. The gLMS scale ranged from ‘‘no sensation’ at 0 to ‘the strongest imaginable sensation of any kind’ at 100. Labels were placed at 1.4 (barely detectable), 6 (weak), 17 (moderate), 35 (strong) and 51 (very strong), respectively. Participants involved in the pilot study did not participate in Experiment One or Two. After the pilot study, four concentrations of ground chilies were chosen for tomato soup, which were selected to evoke barely detectable, low, medium, and high oral burn. Three concentrations of ground chilies were chosen for lunch meals which were likely to provoke no, low and high oral burn intensity. Tomato soup, beef burger patties, and curried rice were chosen as food matrices to represent commonly consumed staple foods in the Netherlands. These foods offer examples of different global cuisines with large sensory differences and facilitated a preliminary comparison of the effect of oral burn on taste, flavor, and mouthfeel across different food forms (liquid/solid) and different textures of solid foods (burger/rice). The preparation procedure of these foods made it possible to control the addition of chili pepper powder to the foods and to ensure homogenous mixing of the test stimulus (ground dried chilies) throughout the different food matrices. Foods were purchased from a local supermarket (Albert Heijn, Wageningen, the Netherlands), including tomato soup (Unox Romige Tomaten Soep; Unilever Nederland B.V., Rotterdam), Indica rice (Pure basmati rice, Tilda), eggs (size M, Albert Heijn), minced beef (16% fat, Albert Heijn), curry paste (Korma, Patak's), coconut milk (Fairtrade Original), salt (NaCl, LoSalt, Klinge Foods) and wheat flour (Albert Heijn).

### 2.3 Experiment 1: tomato soup

#### 2.3.1 Tomato soup preparation

Tomato soup was prepared with a standardized procedure described previously (Lyu et al., 2022). Different amounts of ground chilies (0.01, 0.03, 0.20, or 0.40%) were added to commercially available ready-to-serve creamy tomato soup (Table 1) to correspond to barely detectable, low, medium and high amounts of oral burn. The soup was kept warm in a water bath at 60 °C before serving.

**Table 1:** Overview of foods used in Experiment 1 (tomato soup) and 2 (beef patty and curried rice) with rheological and mechanical properties. Values are reported as mean ± standard deviation.

Experiment 1: 200 g tomato soup						
	Burn level	Barely detectable burn	Low burn	Medium burn	High burn	*p* value
	Ground chilies concentration (w/w %)	0.01	0.03	0.20	0.40	–
	Consistency K (mPa s^n^)	3031	2871	2930	2921	–
	Viscosity at 50 s^−1^ (mPa s)	205 ± 7	202 ± 5	205 ± 6	207 ± 8	0.10
	Flow behavior index n (−)	0.31	0.31	0.31	0.32	–

Experiment 2: 100 g burger patty with 100 g curried rice									
	Burn level	No burn		Low burn		High burn		*p* value (Beef burger)	*p* value (Curried rice)
	Food products	Beef patty	Curried rice	Beef patty	Curried rice	Beef patty	Curried rice	–	–
	Ground chilies concentration (w/w%)	0.0	0.0	0.6	0.4	1.2	1.0	–	–
	Cooking loss (g)	47.0 ± 8.6	–	46.3 ± 6.5	–	47.1 ± 6.9	–	0.85	–
	Instrumental hardness (N)	54 ± 10	28 ± 4	55 ± 10	29 ± 4	55 ± 10	28 ± 5	0.58	0.54

Note: Consistency K and flow behavior index n were obtained by fitting the averaged experimental flow curves in the shear rate range of 1–100 s^−1^ with the Ostwald–de Waele power-law model.

#### 2.3.2 Characterization of rheological properties of soups

Flow curves of tomato soup differing in ground chilies concentrations were recorded using a rheometer (MCR 301, Anton Paar, Graz, Austria) with a concentric single gap cylinder geometry (C-CC17/T200/TI) at 60 °C to determine whether addition of ground chilies influenced rheological properties. Continuous flow measurements were performed by increasing the shear rate in logarithmic steps from 1 s^−1^ to 500 s^−1^ and then decreasing from 500 s^−1^ to 1 s^−1^. Fitting of flow curves with the Ostwald-de Waele power-law model (η = K γ^n−1^) was done in the shear rate range of 1–100 s^−1^ to obtain consistency index *k* and power-law exponent *n* (Aguayo-Mendoza et al., 2019; Lyu et al., 2021). Measurements were done in triplicate.

#### 2.3.3 Rate-All-That-Apply (RATA)

Prior to the first test session, a familiarization session (60 min) was used to acquaint participants (n = 23) with the definitions of sensory terms (Supplementary Materials, Table S1), the RATA method, the use of a 9-point category scale and its anchors, as well as the palate cleansing procedure. After familiarization, participants reported they understood how to perform the RATA test. Before starting the test session, participants were asked again if the procedure was clear and it was explained again if necessary. Data were collected using EyeQuestion software (Version 3.9.7, Logic8 EyeQuestion software).

#### 2.3.4 Oral processing behavior of tomato soups

Participants (n = 23) were invited to attend two test sessions scheduled at least 1 week apart. Participants received two soup samples per session for a total of four samples across the experiment. A 200 g portion of each soup was offered in a plastic bowl with a tablespoon. A 2.5-min break in between samples was enforced using EyeQuestion to minimize acute sensitization and desensitization effects. Sessions were held between 2:00 p.m. and 5:00 p.m. Each session lasted approximately 45 min and started with ratings of hunger (“not hungry at all” to “very hungry”) and fullness (“not full at all” to “very full”) using a 100 mm VAS scale on paper. The soup bowl was placed on a balance (Kern, type PCD 10K0.1, KERN & Sohn GmbH, Germany) concealed under a table so participants did not see the balance. The weight (g) of the soup bowl was continuously recorded by the balance at a 1-s sampling rate. Participants also received a glass of water (∼200 mL) they could consume freely during the session. The presentation order of tomato soup across sessions was counterbalanced over participants to minimize position and carryover effects.

Participants were recorded during soup consumption using a Logitech C270 HD webcam (Logitech SA, Morges, Switzerland) that was positioned ∼30 cm from participants. Participants were instructed to maintain their head straight to the camera, and not to block their mouth or face with their hand while eating. Panelists ate the soups using a spoon until the bowl was empty. Participants were instructed to indicate the main moment of swallowing by raising their hand and videos were annotated post hoc by a researcher using ELAN (Version 6.2) to quantify oral processing behaviors. The frequencies of key point events (bites, chews and swallows) and the duration of a single continuous event (time of food in mouth) were recorded and sip/bite size (g/bite), eating rate (g/min), chews per bite, and total oral exposure time (min) were derived. Measures of oral processing behavior are outlined in Table 2. To ensure consistent annotations, a random selection of 10% of the videos was annotated by a second researcher and results were compared, revealing high correlations.

**Table 2:** Definition of oral processing behavior parameters used for Experiment 1 (tomato soup) and 2 (beef patty and curried rice).

Oral processing parameter	Definition	Experiment 1	Experiment 2
Food intake (g)	Cumulative weight of all bites	X	X
Water intake (g)	Cumulative weight of drinking water	X	
Total consumption time (min)	Total time from the first bite/sip until swallow of the last bite/sip including time between bites/sips	X	X
Total oral exposure time (min)	Cumulative time for all bites from start of each bite until the last swallow of each bite	X	X
Total time between bites/sips (min)	Total time without food in the mouth	X	X
Total number of bites/sips (−)	Number of bites or sips	X	X
Bite/sip size (g)	Food intake (g) divided by total number of bites (−)	X	X
Number of chews per bite (−)	Average number of chews per mouthful/bite		X
Chewing rate (chews/s)	Total number of chews (−) divided by total oral exposure time (s)		X
Eating rate (g/min)	Food intake (g) divided by total consumption time (min)		X
Number of water sips (−)	Number of times participants sip water	X	X
Total number of swallows (−)	Number of times participants raised hand to indicate swallow	X	X
Chewing cycle duration (s)	Dividing the total oral exposure time of food (s) by the number of chews		X

### 2.4 Experiment 2: beef patties with curried rice

#### 2.4.1 Lunch meal preparation

Lunch meals differing in ground chilies concentration were provided: (i) a meal without added ground chilies (no burn), (ii) a meal with a low concentration of added ground chilies (low burn), and (iii) a meal with high concentrations of added ground chilies (high burn). Each meal consisted of a 100 g portion of a beef patty consumed with hands and a 100 g portion of curried rice consumed with a tablespoon. Beef patties were combined with curried rice with a matched concentration of ground chilies (0.0 with 0.0%; 0.6 with 0.4%; 1.2 with 1.0%; for convenience, we refer to these chili concentrations as no, low and high oral burn conditions) and each was consumed as a separate lunch meal (Table 1). Across all meals, a standardized cooking protocol was followed, as described by Lyu et al. (2022). Cooking losses of the patties were calculated from the differences in the weight of patties before and after roasting.

#### 2.4.2 Characterization of mechanical properties of beef patties and curried rice

The hardness of beef patty and curried rice differing in ground chilies concentrations were determined using a texture analyzer (TA-XT plus, Stable Micro Systems with a cylindrical probe (P/75, 75 mm stainless cylinder) to assess the influence of addition of ground chilies to beef patty and curried rice on mechanical properties. Compression tests were performed on beef patty cubes (15 × 15 × 15 mm). For curried rice (5 g) single layer of grains (around 25–30 grains of rice) were placed on the plate of the texture analyzer. Hardness was defined as the peak force (N) required to compress beef patties to 80% strain and curried rice to 90% strain, respectively. Measurements were repeated six times for each sample.

#### 2.4.3 Characterization of sensory properties of beef patties and curried rice

Participants (n = 49) attended three lunch sessions at least 1 week apart. Within a session, participants were asked to fully consume a portion of one meal comprising 100 g beef patty served with 100 g curried rice. Sessions were held between 11:30 a.m. and 1:00 p.m. Each session lasted approximately 45 min and started with ratings of hunger (“not hungry at all” to “very hungry”) and fullness (“not full at all” to “very full”) using a 100 mm VAS scale on paper. Then participants received the lunch meals on a plate. The plate was placed on a balance (Kern, type PCD 10K0.1, KERN & Sohn GmbH, Germany) hidden under a table. The weight of the meal plate was recorded by the hidden balance once per second. Participants also received a glass of water (∼200 mL) they could consume freely during the session. The presentation order of lunch meals across three sessions was counterbalanced over participants to minimize position and carryover effects.

##### 2.4.3.1 Bolus collection and sensory assessment

Participants (n = 49) were instructed to take one bite of the beef patty or curried rice, chew in their usual manner until they would swallow but instead spit out the food into a pre-weighed aluminum dish covered with a lid. After expectoration, participants rated oral burn and hardness intensity of beef patty and curried rice using a general Labelled Magnitude Scale (gLMS) (Hayes et al., 2013) and disliking/liking using a generalized Degree of Liking (gDOL) scale (Byrnes and Hayes, 2013).

Expectorated bolus samples were placed on aluminum dishes, weighed and dried for 16–18 h at 105 °C in an atmospheric oven (Venti-line, VWR®). After drying, samples were cooled in a desiccator for 30 min and weighed. Dry matter content of each sample was measured in triplicate. Saliva content was calculated by subtracting the water content of the food from the water content of the expectorated bolus. Saliva incorporation rate was calculated by dividing saliva incorporated in bolus (g) by oral exposure time of one bite. Calculations assumed the bolus was fully expectorated.

##### 2.4.3.2 Oral processing behavior of beef patties and curried rice

After bolus collection and sensory assessment of the first bite of beef patty and curried rice, participants were instructed to consume the rest of the meal while being video recorded. Oral processing behaviors were annotated from video recordings as described in Experiment One (see Section 2.3.4).

### 2.5 Statistical data analysis

All data were analyzed using IBM SPSS Statistics 25.0 (SPSS Inc., USA). Normality was checked and non-normal data were log-transformed.

#### 2.5.1 Chili pepper questionnaire

The chili pepper questionnaire used in the present study included the following six items: (1) ‘How often do you eat all types of chili peppers in foods including Mexican, Indian, Chinese, and other foods that contain chili pepper and cause tingling or burning?’ (8 category scale: 0 = never, 1 = once a year or less, 2 = less than once per month, 3 = 1–3 times per month, 4 = once a week, 5 = 3–4 times a week, 6 = every day, and 7 = more than once a day); (2) ‘How much do you like the taste of chili peppers in your food?’ (9 point hedonic scale: 1 = dislike extremely to 9 = like extremely); (3) ‘How much do you like the burn of chili peppers in your food?’ same scale as (2); (4) ‘I think chili peppers make food taste better.’ (9 point scale: 1 = Completely disagree to 9 = completely agree); (5) ‘Without hot spices, I find that food tastes too bland.’, same scale as (4); and (6) ‘I find it hard to appreciate the flavors of the food when the food contains hot spices.’ same scale as (4).

Self-reported intake frequency extracted from the overall frequency question (Item 1) was annualized to express estimated consumption frequency on a yearly basis (e.g., less than once per month = 6/year, one to three times per month = 24/year, once a week = 52/year, etc.), as described previously by Byrnes and Hayes (2013). A median split based on annualized chili pepper intake frequency was used to categorize all consumers into infrequent and frequent chili pepper consumers. Independent samples *t*-tests were conducted to compare group differences between infrequent and frequent consumers of chili peppers and spicy foods in scores of the chili pepper questionnaire, burn intensity and disliking/liking ratings.

#### 2.5.2 Oral processing behavior parameters

Oral processing behaviors extracted from the annotation of videos are defined in Table 2. Independent samples *t*-tests were conducted to compare group differences between infrequent and frequent consumers of chili peppers and spicy foods in burn intensity and hedonic ratings, saliva incorporation rate and oral processing parameters. One-way analyses of variance (ANOVAs) were performed separately for cooking loss, instrumental and sensory hardness, burn intensity ratings and hedonic ratings for beef patties and curried rice differing in chili pepper concentrations. ANOVAs were conducted to compare means of all oral processing parameters and saliva content between beef patties differing in chili pepper concentration and between curried rice differing in chili pepper concentration. Pearson's correlation coefficients were calculated for the relationships between sip/bite size and number of sips/bites. A significance level of p < 0.05 was chosen.

## 3 Results

### 3.1 Rheological and mechanical properties of foods

Rheological properties of tomato soup and mechanical properties of beef patty and curried rice are summarized in Table 1. Apparent viscosity at a shear rate of 50 s^−1^ (η_50s-1_) of tomato soups was not significantly affected (p > 0.05) by added ground chilies. Consistency K corresponding to viscosity at a shear rate of 1 s^−1^ and flow behavior index n indicating the magnitude of shear-thinning behavior (0 < n < 1) obtained by fitting the averaged experimental flow curves with the Ostwald-de Waele power-law model were similar across all soups, regardless of ground chilies addition. These findings demonstrate that the rheological properties of the tomato soups were not influenced by adding powdered ground chilies. Similarly, no significant differences (p > 0.05) in instrumental hardness of beef patties and curried rice (Table 1) were observed, indicating the instrumental hardness of beef patties and curried rice were independent of ground chilies addition. These findings are in line with the results of Reinbach et al. (2007) and Lyu et al. (2021). This implies any potential differences in oral processing behaviors between foods differing in ground chilies concentration are caused by the effects of the oral burn on oral processing behavior rather than by changes in the rheological or mechanical properties of the foods.

### 3.2 Participant characteristics

Characteristics of study participants in Experiment One and Experiment Two are summarized in Table 3. Participants in both experiments showed a wide variation in intake frequency with an interquartile range (IQR) of 24–182 times per year and they were categorized as infrequent and frequent chili pepper consumers using a median split (52 times per year) of annualized chili pepper intake frequency. Therefore, participants in Experiment One were categorized into infrequent (n = 12; 11 females) and frequent (n = 11; 7 females) chili pepper consumers, and participants in Experiment Two were categorized into infrequent (n = 25; 18 females) and frequent (n = 24; 13 females) chili pepper consumers. As would be expected, significant differences in mean yearly intake frequency were observed between infrequent and frequent consumers (p < 0.001) in both experiments, indicating the median split resulted in a clear separation of consumers based on their habitual chili pepper use. Frequent chili pepper consumers showed higher hedonic ratings for the taste of chili peppers than infrequent consumers (p < 0.05), consistent with prior work (Lyu et al., 2021; Nolden and Hayes, 2017).

**Table 3:** Characteristics of study participants of Experiment 1 (n = 23) and 2 (n = 49). Infrequent and frequent chili pepper consumers were separated by median split based on their yearly chili pepper intake frequency. Values are reported as mean ± standard deviation. Characteristics with significant differences between intake groups are highlighted in bold.

Experiment	Experiment 1				Experiment 2			
	All participants	Infrequent consumers	Frequent consumers	*p* value	All participants	Infrequent consumers	Frequent consumers	*p* value
(n)	23	12	11	–	49	25	24	–
Age (years)	25.1 ± 3.3	25.2 ± 3.7	25.1 ± 3.3	–	26.2 ± 7.8	27.6 ± 9.8	24.8 ± 5.0	–
BMI (kg/m^2^)	22.6 ± 2.8	22.4 ± 2.9	22.6 ± 2.8	–	21.8 ± 1.8	21.9 ± 1.9	21.6 ± 1.8	–
Yearly chili pepper intake frequency	66.9 ± 5.2	**22.2 ± 4.4**	**222.8 ± 2.0**	**<0.001**	46.4 ± 3.1	**20.2 ± 2.3**	**100.2 ± 2.0**	**<0.001**
Hedonic ratings for tastes of chili peppers	6.9 ± 1.9	**5.9 ± 2.1**	**8.0 ± 0.9**	**0.006**	6.3 ± 1.4	**5.8 ± 1.5**	**6.8 ± 1.1**	**0.01**
Hedonic ratings for burn of chili peppers	5.8 ± 2.0	5.1 ± 2.0	6.5 ± 1.8	0.08	5.4 ± 1.7	**4.8 ± 1.7**	**6.1 ± 1.5**	**0.01**
Hedonic ratings for better tastes with chili peppers	6.4 ± 2.1	**5.5 ± 2.3**	**7.5 ± 1.1**	**0.02**	5.9 ± 2.0	**5.3 ± 1.9**	**6.4 ± 2.0**	**0.04**
Hedonic ratings for food tastes without hot spices	5.7 ± 2.1	**4.8 ± 1.9**	**6.7 ± 1.9**	**0.02**	4.3 ± 2.1	4.1 ± 1.7	4.6 ± 2.5	0.41
Hedonic ratings for flavor of food with hot spices	3.5 ± 2.1	3.9 ± 2.1	3.1 ± 2.1	0.35	4.7 ± 2.1	5.1 ± 2.1	4.3 ± 2.1	0.18
Chili pepper score	32.6 ± 7.2	**28.4 ± 7.0**	**37.2 ± 4.2**	**0.002**	30.3 ± 5.7	**28.0 ± 4.7**	**32.7 ± 5.8**	**0.003**

### 3.3 Experiment 1: tomato soups

#### 3.3.1 Sensory properties of tomato soups (RATA)

Intensities of sensory attributes from the Rate-All-That-Apply [RATA] protocol for tomato soups are shown in Supplementary Materials Table S2. Of the 8 attributes, only oral burn differed significantly across soups; as expected, burn ratings increased with increasing ground chilies concentration (p < 0.001). Mean burn did not differ between the two lowest concentrations, which showed less burn than the medium soup; the highest concentration burned more than the medium soup, suggesting the pilot dose-finding work was generally successful.

#### 3.3.2 Oral processing behaviors of tomato soups

Oral processing behavior of tomato soups differing in ground chilies concentration is summarized in Table 4. No significant differences (p > 0.05) were found between soups differing in oral burn for total food intake, number of sips, average sip size, number of swallows, total consumption duration or total exposure time. Participants consumed 182–188 g of the tomato soups, indicating that participants mostly complied with our instruction to consume the entire bowl (200 g). As expected, increased oral burn significantly increased the number of water sips by 200% [F (3, 88) = 2.91, p = 0.04] and total water intake by 192% [F (3, 88) = 5.51, p = 0.002]. Total time between sips, especially for infrequent chili pepper consumers, significantly increased by 56% with increasing ground chilies concentration [F (3,40) = 4.25, p = 0.01]. Differences in oral processing behaviors between infrequent and frequent chili users were compared (Table 4). Significant differences between intake groups were observed for water intake (t = 2.18, p = 0.01) and number of water sips (t = 2.23, p = 0.01) with infrequent users taking more sips and drinking more water than frequent users. All other oral processing parameters did not differ significantly between infrequent and frequent chili users.

**Table 4:** Oral processing behaviors (n = 23) of tomato soups (Experiment 1) varying in ground chilies concentration. Values are reported as mean ± standard deviation. Parameters with significant differences between infrequent and frequent consumers are highlighted in bold. Different superscript letters indicate significant differences between means (p < 0.05) across samples separately for all participants, infrequent and frequent consumers.

Parameters participants	Ground chilies concentration (%)	Food intake (g)	Total number of sips (−)	Averaged sip size (g)	Number of swallows (−)	Number of water sips (−)	Water intake (g)	Total consumption time (min)	Total oral exposure time (min)	Total time between sips (min)
All participants (n = 23)	0.01	182.0 ± 7.6	22.8 ± 5.2	7.6 ± 1.8	22.8 ± 5.2	**1.1 ± 0.3**^**a**^	**28.9 ± 8.2**^**a**^	3.8 ± 0.9	1.1 ± 0.3	**2.7 ± 1.1**^**a**^
	0.03	188.0 ± 6.9	22.4 ± 4.9	7.9 ± 1.6	22.4 ± 4.9	**1.1 ± 0.3**^**a**^	**31.8 ± 9.8**^**a**^	4.0 ± 1.2	1.1 ± 0.4	**2.9 ± 1.1**^**a**^
	0.20	187.5 ± 9.7	22.0 ± 6.0	8.0 ± 1.8	22.0 ± 6.0	**1.7 ± 0.5**^**ab**^	**37.5 ± 10.2**^**a**^	4.0 ± 1.5	1.0 ± 0.4	**3.0 ± 0.8**^**a**^
	0.40	186.0 ± 8.1	22.4 ± 6.4	8.0 ± 1.7	22.4 ± 6.4	**3.3 ± 1.0**^**b**^	**84.6 ± 15.2**^**b**^	4.1 ± 0.8	1.0 ± 0.4	**3.1 ± 1.3**^**b**^
Infrequent consumers (n = 12)	0.01	185.1 ± 7.0	22.7 ± 8.3	7.4 ± 2.2	22.6 ± 8.2	**2.5 ± 1.8**^**a**^	**61.9 ± 48.4**^**ab**^	3.5 ± 1.4	1.0 ± 0.4	**2.5 ± 0.9**^**a**^
	0.03	187.6 ± 2.0	22.0 ± 7.4	8.0 ± 2.2	22.0 ± 7.4	**1.9 ± 0.7**^**a**^	**61.3 ± 62.4**^**ab**^	3.3 ± 1.2	0.9 ± 0.3	**2.4 ± 1.3**^**a**^
	0.20	187.3 ± 10.0	23.1 ± 9.8	8.2 ± 2.7	23.0 ± 9.7	**3.3 ± 3.2**^**a**^	**57.4 ± 57.4**^**a**^	3.9 ± 0.9	0.9 ± 0.3	**2.5 ± 1.1**^**a**^
	0.40	181.0 ± 13.0	23.3 ± 10.4	8.1 ± 2.3	23.3 ± 10.4	**6.6 ± 6.8**^**b**^	**141.2 ± 68.0**^**b**^	4.2 ± 0.8	0.9 ± 0.2	**3.9 ± 0.5**^**b**^
Frequent consumers (n = 11)	0.01	181.5 ± 19.2	22.9 ± 2.8	7.7 ± 1.6	22.9 ± 2.8	**0.4 ± 0.7**^**a**^	**11.3 ± 17.7**^**a**^	3.9 ± 1.2	1.1 ± 0.3	**2.8 ± 0.8**
	0.03	188.3 ± 8.6	22.6 ± 7.5	7.9 ± 1.3	22.6 ± 3.1	**0.7 ± 1.4**^**a**^	**16.1 ± 27.4**^**a**^	4.3 ± 1.5	1.2. ± 0.4	**3.3 ± 1.2**
	0.20	187.6 ± 14.4	21.4 ± 7.9	7.9 ± 1.3	21.4 ± 2.9	**0.9 ± 1.6**^**a**^	**26.8 ± 42.3**^**ab**^	4.1 ± 0.9	1.1 ± 0.4	**3.6 ± 1.5**
	0.40	188.8 ± 6.2	22.0 ± 8.3	8.0 ± 1.5	22.0 ± 3.3	**1.6 ± 2.4**^**b**^	**54.4 ± 56.4**^**b**^	4.0 ± 0.8	1.1 ± 0.4	**3.7 ± 0.7**

### 3.4 Experiment 2: beef patties and curried rice

#### 3.4.1 Burn intensity and hedonic ratings

As shown in Table 5, burn ratings for beef patties and curried rice increased significantly with increasing ground chilies concentration (beef patty: [F (2,144) = 25.4, p < 0.001]; curried rice: [F (2,144) = 58.8, p < 0.001]). Mean oral burn intensity of beef patties (0.6 and 1.2% ground chilies) and curried rice (0.4% ground chilies) corresponded to moderate burn on a gLMS, while the oral burn intensity of curried rice (1.0% ground chilies) corresponded to a strong burn intensity. Even though the concentration of ground chilies was higher in beef patties than in curried rice, the perceived burn from curried rice was greater than for the beef patties, highlighting a possible effect of food matrix. Mean hedonic ratings for beef patties differing in ground chilies concentration showed no significant differences [F (2,144) = 0.35, p = 0.71]. Mean hedonic ratings for curried rice decreased as ground chilies concentration increased to 1.0% [F (2,144) = 4.76, p < 0.01].

**Table 5:** Averaged ratings (n = 49) of burn intensity from general Labelled Magnitude Scale (gLMS) and disliking/liking from generalized Degree of Liking scale (gDOL) of beef patty and curried rice with different ground chilies concentrations. Values are reported as mean ± standard deviation. Different superscript letters indicate significant differences between means (p < 0.05) across samples separately for all participants, infrequent and frequent consumers. Ratings with significant differences between infrequent and frequent consumers are highlighted in bold.

		No burn		Low burn		High burn	
	Consumers	Beef patty (0.0% chilies)	Curried rice (0.0% chilies)	Beef patty (0.6% chilies)	Curried rice (0.4% chilies)	Beef patty (1.2% chilies)	Curried rice (1.0% chilies)
Burn intensity (gLMS)	All (n = 49)	2.7 ± 1.3^a^	2.6 ± 1.6^a^	18.9 ± 2.0^b^	31.8 ± 2.3^b^	26.3 ± 2.7^c^	40.9 ± 2.9^b^
	Infrequent (n = 25)	2.2 ± 1.2^a^	2.6 ± 1.3^a^	20.2 ± 2.2^a^	**35.5 ± 2.5**^**a**^	**30.9 ± 2.8**^**a**^	**46.7 ± 3.3**^**a**^
	Frequent (n = 24)	3.2 ± 1.5^a^	3.0 ± 2.0^a^	17.5 ± 1.7^b^	**28.6 ± 2.0**^**a**^	**21.6 ± 2.4**^**b**^	**35.8 ± 2.1**^**a**^
Disking/Liking (gDOL)	All (n = 49)	32.8 ± 4.6^a^	18.3 ± 3.7^a^	29.7 ± 4.9^a^	18.8 ± 4.2^a^	27.1 ± 5.0^a^	1.4 ± 5.5^b^
	Infrequent (n = 25)	32.2 ± 4.2^a^	19.4 ± 3.2^a^	32.6 ± 3.5^a^	**11.7 ± 4.5**^**a**^	23.5 ± 4.6^a^	**−7.5 ± 5.2**^**a**^
	Frequent (n = 24)	33.3 ± 5.0^a^	17.2 ± 4.2^a^	26.8 ± 6.1^a^	**25.9 ± 3.7**^**b**^	30.6 ± 5.4^a^	**10.4 ± 5.6**^**b**^
Sensory hardness	All (n = 49)	5.0 ± 1.5^a^	3.7 ± 1.9^a^	5.1 ± 1.5^a^	3.6 ± 1.8^a^	4.9 ± 1.6^a^	3.6 ± 1.7^a^
	Infrequent (n = 25)	4.9 ± 1.7^a^	4.1 ± 1.8^a^	4.8 ± 1.5^a^	3.6 ± 1.5^a^	4.4 ± 1.5^a^	3.5 ± 1.4^a^
	Frequent (n = 24)	5.2 ± 1.2^a^	3.7 ± 1.9^a^	5.5 ± 1.4^a^	3.8 ± 1.9^a^	5.5 ± 1.5^a^	3.8 ± 1.7^a^

Table 5 summarizes differences in mean burn intensity and hedonic ratings between infrequent and frequent consumers. For burn, significant differences between intake groups were observed for beef patties (1.2% ground chilies: p = 0.02) and curried rice (0.4% ground chilies: p = 0.03; 1.0% ground chilies: p = 0.02) with frequent chili pepper consumers (n = 24) reporting less burn than infrequent consumers (n = 25), as expected. For hedonic ratings, no significant differences were observed between intake groups for the beef patties; conversely, hedonic ratings for the curried rice did show group differences, with frequent consumers having higher liking scores than infrequent consumers. The magnitude of the differences in liking between the groups changed with ground chilies concentration. Specifically, differences in liking between infrequent and frequent consumers increased as the concentration of capsaicin increased. Significant differences in liking between infrequent and frequent chili pepper consumers were observed at 0.4% and 1.0% of ground chilies (0.4% ground chilies: t = 2.01, p = 0.02; 1.0% ground chilies: t = 2.01, p < 0.001) with frequent chili pepper consumers (n = 24) reporting higher hedonic ratings compared to infrequent consumers (n = 25).

#### 3.4.2 Oral processing behaviors of beef patties and curried rice

Oral processing behaviors of the lunch meals varying in ground chilies concentrations are summarized in Table 6. In order to check for compliance of participants who were instructed to consume all foods offered (100 g curried rice and 100 g beef patty), intake of the fixed portion meal was checked. Intake did not vary with ground chilies concentration for the beef patty or curried rice. On average, participants consumed 96–99 g of the beef patty [F (2,147) = 0.25, p = 0.78] and 93–96 g of the curried rice [F (2,147) = 0.016, p = 0.85], demonstrating that participants followed our instructions to consume all the provided food. There was no evidence of an effect on total meal duration, as participants consumed each meal in ∼14 min [F (2,147) = 0.41, p = 0.66]. However, total oral exposure time significantly decreased by ∼22% with increasing ground chilies concentration (F (2,147) = 5.7, p < 0.01). Total time between bites also increased by ∼88% with increasing ground chilies concentration [F (2,147) = 6.04, p < 0.01]. Total number of water sips increased by ∼122% with ground chilies addition [F (2,147) = 8.36, p < 0.001].

**Table 6:** Oral processing behaviors (n = 49) during meal intake (Experiment 2: 100 g beef patty and 100 g curried rice were offered together), and total consumption time, total oral exposure time, total time between bites and total number of water sips of lunch meals varying in ground chilies concentration. Values are reported as mean ± standard deviation. Different superscript letters indicate significant (p < 0.05) differences between means within a food category.

	No burn		Low burn		High burn	
Lunch meal	Beef patty + curried rice		Beef patty + curried rice		Beef patty + curried rice	
Food intake (g)	194.7 ± 21.5^a^		192.4 ± 28.8^a^		191.0 ± 29.3^a^	
Total consumption time (min)	13.5 ± 2.3^a^		13.4 ± 6.7^a^		14.3 ± 7.2^a^	
Total oral exposure time (min)	8.2 ± 3.4^a^		7.1 ± 2.6^a^		6.4 ± 2.2^b^	
Total time between bites (min)	4.2 ± 3.1^a^		6.1 ± 2.5^b^		7.9 ± 4.0 ^C^	
Total number of water sips (−)	5.5 ± 3.5^a^		8.4 ± 5.9^b^		12.2 ± 12.5^c^	
Meal component	Beef patty	Curried rice	Beef patty	Curried rice	Beef patty	Curried rice
Food intake (g)	97.8 ± 16.5^a^	96.5 ± 11.9^a^	97.9 ± 16.1^a^	94.1 ± 16.4^a^	97.1 ± 18.4^a^	93.5 ± 17.7^a^
Total number of bites (−)	8.2 ± 2.4^a^	12.1 ± 3.1^a^	8.9 ± 2.8^a^	12.0 ± 0.5^a^	8.9 ± 2.9^a^	12.1 ± 3.5^a^
Bite size (g)	12.5 ± 3.0^a^	8.4 ± 1.9^a^	11.8 ± 3.4^a^	8.5 ± 2.5^a^	12.0 ± 4.0^a^	8.1 ± 2.0^a^
Total number of chews (−)	203.7 ± 101.0^a^	174.2 ± 83.3^a^	172.7 ± 108.6^a^	140.3 ± 8.9^b^	143.2 ± 89.6^b^	132.4 ± 70.8^c^
Oral exposure time (min)	4.2 ± 1.9^a^	4.0 ± 1.9^a^	3.9 ± 1.7^a^	3.1 ± 1.1^b^	3.3 ± 1.4^b^	3.0 ± 1.2^b^
Eating rate (g/min)	27.5 ± 11.5^a^	30.5 ± 16.4^a^	29.9 ± 10.6^a^	33.4 ± 11.9^a^	32.7 ± 12.7^a^	35.3 ± 13.7^a^
Number of chews per bite (−)	21.5 ± 10.8^a^	14.6 ± 7.9^a^	19.4 ± 10.0^a^	12.4 ± 5.4^b^	16.4 ± 8.8^b^	10.9 ± 5.2^c^
Chewing rate (chews/s)	0.7 ± 0.4^a^	0.8 ± 0.4^a^	0.8 ± 0.4^a^	0.8 ± 0.4^a^	0.7 ± 0.4^a^	0.8 ± 0.4^a^
Chewing cycle duration (s)	1.7 ± 0.8^a^	1.6 ± 0.7^a^	1.6 ± 0.7^a^	1.5 ± 0.6^a^	1.7 ± 0.7^a^	1.6 ± 0.7^a^
Total numbers of swallows (−)	13.4 ± 6.5^a^	14.7 ± 5.4^a^	14.2 ± 6.1^a^	14.2 ± 5.4^a^	13.8 ± 6.6^a^	15.1 ± 5.6^a^
Total number of water sips (−)	2.6 ± 1.8^a^	2.9 ± 2.3^a^	4.3 ± 3.5^a^	4.1 ± 3.2^a^	6.7 ± 7.7^b^	5.6 ± 6.8^b^

As shown in Table 6, significant effects of oral burn on total number of chews (beef patty: F (2,147) = 3.82, p = 0.02; curried rice: F (2, 147) = 9.12, p < 0.001), number of chews per bite (beef patty: F (2,147) = 3.83, p = 0.03; curried rice: F (2, 147) = 4.12, p = 0.02), and oral exposure time (beef patty: F (2,147) = 3.19, p = 0.04; curried rice: F (2, 147) = 6.16, p = 0.003) were found for both meal components. With increasing ground chilies concentration, total number of chews decreased by 30% for beef patties (F (2,147) = 4.31, p = 0.01) and by 24% for curried rice (F (2,147) = 4.52, p = 0.01), number of chews per bite significantly decreased by 24% for beef patties (F (2,147) = 4.61, p = 0.01) and by 25% for curried rice (F (2,147) = 4.61, p = 0.01), oral exposure time decreased by 21% for beef patties (F (2,147) = 3.54, p = 0.03) and by 25% for curried rice (F (2,147) = 6.16, p = 0.002). Conversely, no significant effects were observed for number of bites, bite size, chewing rate, eating rate, number of swallows and chewing cycle duration for the beef patties or curried rice. No significant differences in oral processing parameters were found between frequent and infrequent consumers of hot foods for beef patties and curried rice (Supplementary Materials Table S3), with two exceptions: the total numbers of swallows and total number of water sips of curried rice with 1.0% chili which differed significantly between intake groups.

**Fig. 1:**
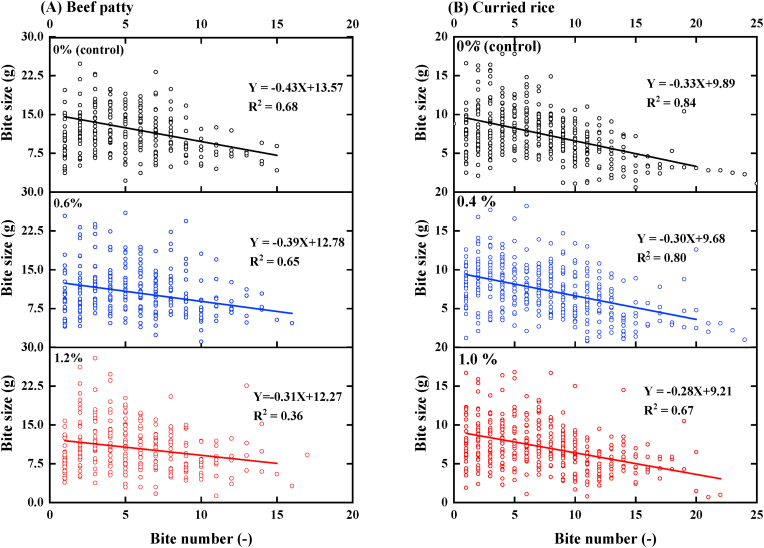
Bite size (n = 49) during consumption of beef patty (A) and curried rice (B) differing in ground chilies concentration as a function of number of bites. The solid lines depict linear regression lines.

Fig. 1 shows bite size from the first to the last bite for beef patties and curried rice differing in ground chilies concentrations. Bite size was negatively related to number of bites, indicating participants reduced bite size from the beginning of the meal to the end. No significant difference in slope of the regression line was found for beef patty [F (2, 831) = 0.18, p = 0.83] and curried rice [F (2,1152) = 1.85, p = 0.16], suggesting oral burn had no effect on the adaptation of bite size during the course of a meal.

**Fig. 2:**
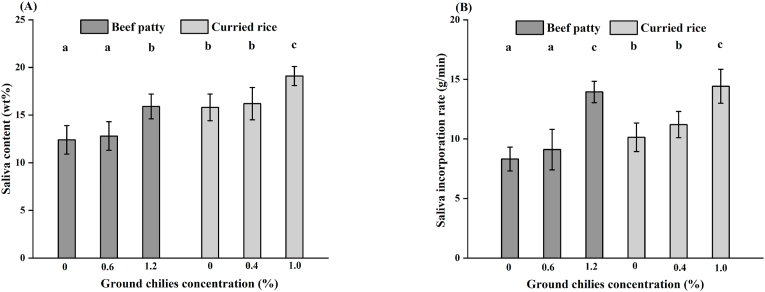
Bolus saliva content (n = 49) at the moment of swallowing (A) and saliva incorporation rate (B). Error bars represent the standard error of the mean. Different letters indicate significant differences between means across product categories. (p < 0.05).

Fig. 2A shows the mean saliva content at the moment of swallowing in beef patty and curried rice bolus. Bolus saliva content significantly increased with increasing concentration of ground chilies for beef patty [F (2,156) = 7.19, p < 0.01] and curried rice [F (2, 156) = 6.34, p < 0.01]. Saliva content increased from 12.4% for beef patties without ground chilies to 13.9% for beef patties with 1.2% ground chilies. Saliva content increased from 15.8% for curried rice without ground chilies to 19.1% for curried rice with 1.0% ground chilies. As shown in Fig. 2B, the rate of saliva incorporation in the bolus increased significantly with increasing ground chilies concentration for curried rice [F (2, 144) = 6.72, p < 0.01] and beef patty [(F (2, 144) = 4.50, p = 0.02].

## 4 Discussion

Many studies have investigated how oral processing behaviors are affected by (a) the physical-chemical properties of foods, (b) consumer characteristics, and (c) taste and aroma intensity – however, little attention has been paid to how chemesthetic sensations might affect oral processing behaviors. Here, we studied how oral burn caused by ground chilies might affect oral processing behaviors for liquid (tomato soups) and solid (beef patties and curried rice) foods. In addition, potential differences in oral processing behaviors between infrequent and frequent chili pepper consumers were explored.

Oral burn intensity increased, as expected, for all foods when ground dried chilies were added (Table 5 and Supplementary Material Table S2). Comparing the two solid foods (beef patty versus curried rice), beef patties had a lower burn intensity than curried rice even when the concentration of added ground chilies was higher (Table 5). It is known that burn intensity decreases when capsaicin is added to fat-containing foods since hydrophobic capsaicin can remain in the oil phase rather than dissolve in saliva. Findings summarized in Table 3, Table 4 suggest that incorporating fat into foods containing capsaicin can reduce perceived burn intensity (Baron and Penfield, 1996; Bolliet et al., 2016; Carden et al., 1999; Hutchinson et al., 1990; Lawless et al., 2000; Lee and Kim, 2013). More recent work also suggests capsaicin may interact with protein (Farah et al., 2022). Participants’ hedonic ratings for curried rice decreased presumably because the amount of heat exceeded their optimum, which was in line with prior work that suggests liking rises to a hedonic optimum and then falls as concentration increases (Lyu et al., 2021; Nolden and Hayes, 2017).

Perceived and instrumental hardness of the beef patty and curried rice was not affected by the addition of chilies; likewise, instrumental measures and perceived texture did not vary for the tomato soups. Nor were any other sensory attributes, besides oral heat, affected by chili addition. It is worth noting there is some controversy on the cross-modal effects of capsaicin/chili on taste, aroma and texture perception. Multiple studies have reported that oral burn suppressed the intensity of gustatory, olfactory and texture sensations (Gilmore and Green, 1993; Green and Hayes, 2003; Karrer and Bartoshuk, 1995; Lawless et al., 1985; Lawless and Stevens, 1984; Lyu et al., 2021; Prescott and Stevenson, 1995; Simons et al., 2002, 2003), while other studies demonstrated that capsaicin had no impact on or even increased gustatory, olfactory and texture perception in simple model stimuli (Frasnelli et al., 2009; Narukawa et al., 2011; Prescott and Stevenson, 1995; Wang et al., 2022; Yang et al., 2021). Thus, further investigations, especially using real, commercially available foods and meals, are needed to deepen our understanding of the cross-modal effects of oral burn on flavor and texture perception.

For beef patties and curried rice, oral exposure time, total number of chews and number of chews per bite decreased significantly with increasing burn intensity while total time between bites increased significantly with increasing burn intensity, while total consumption time of tomato soups and meals composed of beef patties and curried rice were not affected (Table 6). These effects demonstrate that consumers adapt their oral processing behaviors to the oral burn intensity experienced during consumption. It seems that consumers’ adaptation of their mastication behavior to mitigate any potential discomfort perceived at higher chili concentrations. Consumers chewed less per bite to reduce oral exposure time while also increasing the time between bites and consuming more water between bites. When combined, these offsetting effects compensated one another such that total consumption time was not affected by oral burn. Despite this, it is worth noting that both beef patties and curried rice were generally liked (Table 5) and almost all foods offered were consumed in their entirety (Table 6). These results are in disagreement with a recent study that observed no significant effects of oral burn on oral processing behaviors of pork meats to which hot sauces were added (Djekic et al., 2021). Our findings indicate that increasing oral burn intensity led to a longer mastication time and a decrease in eating rate. The lack of statistical significance of that study might be caused by a limited power (10 participants assessing single bites in triplicate, for 30 observations per food) whereas our study of solid foods used 49 participants eating meals (20 bites on average), resulting in 980 observations per sample. Another major difference between studies is that in our approach participants consumed a meal with multiple bites, so it is a more realistic consumption context that allows us to characterize time between bites and observe adaptation in oral behavior over the course of the consumption. However, for the tomato soups we did not observe that oral exposure time and total time between sips were altered by increased burn intensity. One possible explanation is that oral exposure time of tomato soup was too short to observe alternations as total oral exposure time of soups was approximately 1 min while total oral exposure time of beef patty and curried rice was longer (∼3 to 4 min).

Greater burn intensity increased number of water sips and water intake for all three foods (tomato soup, beef patties and curried rice), as shown in Table 4, Table 6. It is interesting to note that the total number of water sips at the lowest burn intensity was 1.1 sips for soups and 5.5 sips for the solid foods, while the total number of water sips at the highest burn tripled to 3.3 sips for soups and doubled to 12.2 sips for the solid foods. The increase in water sips and water intake can be explained by participants’ attempts to use water to decrease discomforting burn sensations. It has been shown that water or other beverages (e.g. milk, beer and cola) may help mitigate the perceived burn through a variety of psychological, physiological, or physical mechanisms (Farah et al., 2022; Nasrawi and Pangborn, 1990; Nolden and Hayes, 2017; Nolden et al., 2019). Even though sip/bite size was negatively related to the number of sips/bites taking in the course of a meal, so from first to last sip/bite (Fig. 1), further statistical analysis demonstrated that number of bites/sips and averaged bite/sip size was not influenced by increased burn sensation. Previous studies investigated the effect of taste and aroma intensity on bite size, suggesting higher taste and aroma intensities resulted in significantly smaller bite size and fewer bites (Bolhuis et al., 2011, 2012; de Wijk et al., 2012; Neyraud et al., 2005). Present results fail to confirm our hypothesis that sip/bite size decreases with burn sensation. de Wijk et al. (2012) proposed a possible explanation for reduced bite size that consumers can self-regulate their sensations via bite size when sensations become more intense, whereby weak sensations were intensified via larger bite sizes and stronger sensations were weakened via smaller bite sizes. In the present study, however, we failed to observe that consumers adapt their bite size to the increased burn sensation of foods as well as a stronger burn sensation over multiple sips or bites. It should be noted that the beef patties were consumed by hand without cutlery, so for the beef patties we can exclude unit size effects introduced by cutlery. One possible explanation, which would require further investigation, is that bite size may be approximately stable over multiple bites, whereby deviations on individual bites are compensated by subsequent bites.

Bolus saliva content significantly increased with increasing concentration of ground chilies (Fig. 2) although number of chews per bite and oral exposure time decreased. These results were consistent with several previous studies (Cowart, 1987; Lyu et al., 2021; Törnwall et al., 2012). Our results suggest that the stimulation of salivation by ground chilies was therefore larger than the decrease in salivation caused by a reduced mastication time which reduced mechanical stimulation of salivation. Similarly to our results, Djekic et al. (2021) compared the effects of three different hot sauces on saliva incorporation in pork meat bolus and found a significant increase in saliva incorporation with the hottest sauce. These findings are in line with previous studies that suggested that trigeminal stimulation by capsaicin induces salivation by modulating the paracellular pathway in salivary glands (Kanehira et al., 2011, 2014; Nasrawi and Pangborn, 1990; Yang et al., 2020). It seems to be controversial whether salivary flow stimulated by chili pepper depends on intake frequency of chili pepper. Wang et al. (2016) suggested that salivation of submandibular and parotid glands stimulated by chili (capsaicin) was independent of daily intake of chili. Nasrawi and Pangborn (1990) found that eaters and non-eaters of chili peppers did not differ significantly in stimulated salivary flow, while other studies reported salivary flow to irritation of chili pepper decreased with increased liking of chili pepper (Rozin et al., 1981). Thus, further research is needed to determine the relationship between saliva production stimulated by chili pepper and intake frequency of chili pepper.

Our study demonstrates that consumers adapt their oral behaviors to the oral burn experienced during consumption of portions of soups and (small) meals of beef patties and curried rice. Notably, these changes are independent of their habitual intake of hot spicy foods. Frequent consumers perceived the oral burn intensity of the solid foods as lower than infrequent consumers, in agreement with previous studies. Despite this, the differences in intensity perception of oral burn between infrequent and frequent consumers did result in differences between these groups in oral behaviors, suggesting that the perceptual differences might be too small to cause behavioral differences. Oral exposure time decreased and time between bites increased with increasing oral burn intensity. This demonstrates that manipulations of the hotness or spiciness of foods are effective in adapting oral behaviors. Future studies should explore the impact of these manipulations on food and energy intake.

## 5 Conclusions

The present study represents a step towards understanding the effects of burn sensations on oral processing behavior, as our results demonstrate that increased burn intensity in liquid foods (tomato soups) increased number of water sips, water intake and total time between sips. Increased burn intensity in solid food (beef patties and curried rice) increased not only total time between bites and total number of water sips but also decreased oral exposure time, total number of chews and number of chews per bite while total consumption time remained unchanged. Saliva content and rate of saliva incorporation into the food bolus of beef patties and curried rice increased with increasing ground chilies concentration. We conclude that consumers adapt their oral processing behaviors to the oral burn intensity of solid foods (beef patties and curried rice) by reducing oral sensory exposure time, increasing time between bites, chewing bites less and consuming more water probably to mitigate the discomfort associated with the burn imparted by ground chilies.

## CRediT authorship contribution statement:

Cong Lyu: Data curation, Methodology, Formal analysis, Investigation, Validation, Writing - original draft, Writing - review & editing. Vonk Marlotte: Formal analysis, Methodology, Investigation, Validation. John E. Hayes: Conceptualization, Writing - review & editing. Jianshe Chen: Conceptualization, Writing - review & editing. Ciarán G. Forde: Conceptualization, Supervision, Writing - review & editing. Markus Stieger: Conceptualization, Supervision, Writing - review & editing.

## Declaration of competing interest

The authors declare that they have no known competing financial interests or personal relationships that could have appeared to influence the work reported in this paper.

## Data Availability

The authors do not have permission to share data.
