# Supplementary material for: The heat is on: Consumers modify their oral processing behavior when eating spicy foods
Source: Curr Res Food Sci. 2023 Sep 29;7:100597. doi: 10.1016/j.crfs.2023.100597 (PMC10569983; doi:10.1016/j.crfs.2023.100597)
Supplement: Multimedia component 1 [file mmc1.docx]

**Table S1.** Sensory attributes and definitions used during the RATA evaluation of tomato soup.

| **Sensory attribute** | **Definition** |
| --- | --- |
| **Flavor/Taste** |  |
| Tomato flavor | Distinctive taste of tomato |
| Sweetness | Sensation of basic sweet taste |
| Sourness | Sensation of basic sour taste |
| Saltiness | Sensation of basic salty taste |
| Overall flavor | Overall flavor perceived in the mouth |
| **Trigeminal** |  |
| Burn | Total intensity of oral burn perceived in mouth and throat. |
| **Mouth-feel** |  |
| Thickness | Ease to deform the food between tongue and palate and perceived resistance to flow |
| Creaminess | Sensation of thick, smooth, velvety mouth-feel |

**Table S2.** Perceived intensities of sensory attributes (Rate-All-That-Apply [RATA], n = 23) of tomato soup. Mean values are shown with standard deviation. Attributes with significant differences between samples are highlighted in bold. Samples not sharing superscript letters are significantly different (p < 0.05).

| Attribute | Tomato soup | | | |  |
| --- | --- | --- | --- | --- | --- |
| Oral burn sensation | Barely detectable | Low | Medium | High | p-value |
| Tomato flavor | 6.3 ± 1.6 | 6.4 ± 1.5 | 6.1 ± 2.0 | 6.2 ± 1.9 | 0.93 |
| Saltiness | 4.0 ± 1.8 | 3.9 ± 1.8 | 3.9 ± 2.0 | 3.3 ± 1.9 | 0.47 |
| Sweetness | 4.3 ± 2.3 | 3.6 ± 2.1 | 3.7 ± 2.2 | 3.4 ± 2.1 | 0.44 |
| Sourness | 4.1 ± 2.1 | 4.3 ± 2.5 | 4.3± 2.2 | 3.7 ± 2.2 | 0.72 |
| **Burn** | **0.3 ± 0.7^a^** | **1.1 ± 1.5^a^** | **3.1 ± 2.3^b^** | **5.3 ± 1.8^c^** | **< 0.001** |
| Thickness | 5.1 ± 2.4^a^ | 5.2 ± 2.2^a^ | 4.8 ± 2.2^a^ | 4.4 ± 2.2^a^ | 0.53 |
| Creaminess | 5.5 ± 2.0^a^ | 5.4 ± 1.9^a^ | 5.1 ± 2.1^a^ | 4.8 ± 1.9^a^ | 0.55 |
| Overall flavor | 6.4 ± 1.5^a^ | 6.3 ± 1.7^a^ | 6.1 ± 2.0^a^ | 5.5 ± 2.0^a^ | 0.23 |

## **Table S3.** Oral processing behaviors (n=49) during meal intake (100g beef patty and 100g curried rice were offered together), and total consumption time, total oral exposure time, total time between bites and total number of water sips of lunch meals varying in ground chilies concentration. Values are reported as mean ± standard deviation. Different superscript letters indicate significant differences between means (p < 0.05) across samples separately for all participants, infrequent and frequent consumers. Parameters with significant differences between infrequent and frequent consumers are highlighted in bold.

|  | **No burn** | | | | **Low burn** | | | | **High burn** | | | |
| --- | --- | --- | --- | --- | --- | --- | --- | --- | --- | --- | --- | --- |
| Lunch meal | Beef patty+curried rice | | | | Beef patty+curried rice | | | | Beef patty+curried rice | | | |
| Consumers | Infrequent | | Frequent | | Infrequent | | Frequent | | Infrequent | | Frequent | |
| Food intake (g) | 188.7 ± 25.1 ^a^ | | 198.1 ± 15.6 ^a^ | | 186.1 ± 36.7 ^a^ | | 198.2 ± 16.5 ^a^ | | 185.5 ± 37.7 ^a^ | | 196.0 ± 16.7 ^a^ | |
| Total consumption time (min) | 13.3 ± 4.0 ^a^ | | 13.5 ± 4.0 ^a^ | | 13.2 ± 3.5 ^a^ | | 13.5 ± 5.0 ^a^ | | 14.3 ± 4.9 ^a^ | | 14.3 ± 4.4 ^a^ | |
| Total oral exposure time (min) | 7.8 ± 3.5 ^a^ | | 8.5 ± 3.4 ^a^ | | 6.7 ± 2.4 ^b^ | | 7.2 ± 2.7 ^b^ | | 6.2 ± 1.9 ^c^ | | 6.4 ± 2.2 ^c^ | |
| Total time between bites (min) | 4.1 ± 3.1^a^ | | 4.2 ± 3.1 ^a^ | | 6.0 ± 2.5 ^b^ | | 6.1 ± 2.5^b^ | | 7.8 ± 4.0^c^ | | 7.9 ± 4.0 ^c^ | |
| Total number of water sips (-) | 5.7 ± 3.9^a^ | | 5.3 ± 3.1 ^a^ | | 9.3 ± 6.7 ^b^ | | 7.5 ± 5.0 ^b^ | | 14.9 ± 15.2 ^c^ | | 9.5 ± 8.8 ^c^ | |
| Meal component | Beef patty | | Curried rice | | Beef patty | | Curried rice | | Beef patty | | Curried rice | |
| Consumers | Infrequent | Frequent | Infrequent | Frequent | Infrequent | Frequent | Infrequent | Frequent | Infrequent | Frequent | Infrequent | Frequent |
| Food intake (g) | 96.1 ± 18.7^a^ | 97.6 ± 13.2^a^ | 94.3 ± 16.0^a^ | 98.8 ± 4.1^a^ | 97.7 ± 19.4^a^ | 98.2 ± 11.8^a^ | 90.4 ± 21.8^a^ | 98.0 ± 5.8^a^ | 96.0 ± 22.6^a^ | 98.3 ± 13.1^a^ | 89.5 ± 22.7^a^ | 97.7 ± 9.1^a^ |
| Total number of bites (-) | 8.0 ± 2.0 ^a^ | 8.4 ± 2.7 ^a^ | 12.1 ± 3.4 ^a^ | 12.0 ± 2.8 ^a^ | 8.8 ± 2.9 ^a^ | 9.0 ± 2.7 ^a^ | 11.7 ± 4.2 ^a^ | 12.3 ± 3.6^a^ | 9.1 ± 2.8 ^a^ | 8.7 ± 3.0 ^a^ | 11.4 ± 3.6 ^a^ | 13.0 ± 3.2 ^a^ |
| Bite size (g) | 12.3 ± 3.0 ^a^ | 12.7 ± 3.1 ^a^ | 8.1 ± 1.8 ^a^ | 8.6 ± 2.1 ^a^ | 11.6 ± 3.3 ^a^ | 12.0 ± 3.5 ^a^ | 8.3 ± 2.6 ^a^ | 8.6 ± 2.4 ^a^ | 11.4 ± 4.2 ^a^ | 12.5 ± 3.8 ^a^ | 8.2 ± 2.0 ^a^ | 7.9 ± 1.9 ^a^ |
| Total number of chews (-) | 172.2±112.9^a^ | 177.1±89.3^a^ | 156.6±78.9 ^a^ | 178.3±88.0 ^a^ | 185.5±128.2 ^a^ | 159.4±84.4 ^a^ | 140.3±69.5 ^a^ | 140.4±56.8 ^a^ | 160.2±107.2 ^a^ | 125.5±64.1 ^a^ | 127.6±80.7 ^a^ | 137.4±60.2 ^a^ |
| Oral exposure time (min) | 4.1 ± 2.0 ^a^ | 4.2 ± 1.8^a^ | 3.7 ± 1.9 ^a^ | 4.2 ± 1.9 ^a^ | 3.8 ± 1.8 ^a^ | 3.9 ± 1.7 ^a^ | 2.9 ± 1.0 ^a^ | 3.3 ± 1.2 ^a^ | 3.5 ± 1.6 ^a^ | 3.1 ± 1.2 ^a^ | 2.7 ± 1.1 ^a^ | 3.3 ± 1.2 ^a^ |
| Eating rate (g/min) | 27.6± 13.0^a^ | 27.5 ± 10.0^a^ | 32.6 ± 18.2^a^ | 28.3 ± 14.3^a^ | 30.0 ± 11.5 ^a^ | 29.9 ± 9.8 ^a^ | 33.6 ± 12.9 ^a^ | 33.3 ± 10.9 ^a^ | 31.1 ± 13.2 ^a^ | 34.4 ± 12.1 ^a^ | 36.7 ± 14.2 ^a^ | 33.8 ± 13.2 ^a^ |
| Number of chews per bite (-) | 21.0 ± 10.3 ^a^ | 22.2 ± 11.5^a^ | 13.7 ± 7.7 ^a^ | 15.5 ± 8.3^a^ | 20.1 ± 9.5 ^a^ | 18.7 ± 10.5 ^a^ | 13.0 ± 6.4 ^a^ | 11.7 ± 4.3 ^a^ | 16.9 ± 8.1 ^a^ | 15.9 ± 9.5 ^a^ | 10.9 ± 5.5 ^a^ | 10.9 ± 4.9 ^a^ |
| Chewing rate (chews/s) | 0.7 ± 0.4 ^a^ | 0.7 ± 0.3 ^a^ | 0.8 ± 0.4 ^a^ | 0.8 ± 0.3 ^a^ | 0.8 ± 0.4 ^a^ | 0.7 ± 0.4 ^a^ | 0.8 ± 0.4 ^a^ | 0.8 ± 0.4 ^a^ | 0.7 ± 0.4 ^a^ | 0.7 ± 0.4 ^a^ | 0.8 ± 0.4 ^a^ | 0.8 ± 0.4 ^a^ |
| Chewing cycle duration (s) | 1.8 ± 0.9 ^a^ | 1.6 ± 0.6 ^a^ | 0.8 ± 0.7 ^a^ | 1.6 ± 0.6 ^a^ | 1.6 ± 0.7 ^a^ | 1.7 ± 0.7 ^a^ | 1.4 ± 0.6 ^a^ | 1.6 ± 0.7 ^a^ | 1.6 ± 0.7 ^a^ | 1.8 ± 0.7 ^a^ | 1.6 ± 0.7 ^a^ | 1.6 ± 0.7 ^a^ |
| Total numbers of swallows (-) | 13.1 ± 6.0^a^ | 13.8 ± 7.2 ^a^ | 13.8 ± 4.8 ^a^ | 15.6 ± 5.9 ^a^ | 14.1 ± 6.3 ^a^ | 14.3 ± 6.2 ^a^ | 13.4 ± 5.4 ^a^ | 15.0 ± 5.3 ^a^ | 14.0 ± 6.9 ^a^ | 13.5 ± 6.3 ^a^ | **13.4 ± 5.8** ^a^ | **16.8 ± 4.9** ^a^ |
| Total number of water sips (-) | 2.8 ± 1.8 ^a^ | 2.4 ± 1.8 ^a^ | 2.9 ± 2.4 ^a^ | 2.8 ± 2.2 ^a^ | 4.9 ± 4.1 ^a^ | 3.7 ± 2.8 ^a^ | 4.4 ± 3.5 ^a^ | 3.9 ± 2.9 ^a^ | 7.3 ± 7.3 ^a^ | 6.1 ± 8.1 ^a^ | **7.6 ± 8.7** ^a^ | **3.4 ± 2.9** ^a^ |
